# Causes of Morbidity and Mortality among Neonates and Children in Post-Conflict Burundi: A Cross-Sectional Retrospective Study

**DOI:** 10.3390/children5090125

**Published:** 2018-09-08

**Authors:** Imelda K. Moise

**Affiliations:** 1 Department of Geography and Regional Studies, College of Arts and Sciences, University of Miami, Coral Gables, FL 33124, USA; moise@miami.edu; Tel.: +1-305-284-2360; 2 Department of Public Health Sciences, Miller School of Medicine, University of Miami, Miami, FL 33136, USA

**Keywords:** neonatal, children, mortality, morbidity, deaths, Sub-Saharan Africa, under-five, incidence, Burundi

## Abstract

The risk of a child dying before age five in Burundi is almost 1.6 times higher than that in the World Health Organization (WHO) African region. However, variations in the all-cause mortality rates across Burundi have not yet been measured directly at subnational levels, age group and by gender. The objective of this study was to describe the main causes of hospitalization and mortality in children during the neonatal period and at ages 1 to 59 months, for boys and girls, and to assess the total annual (2010) burden of under-five morbidity and mortality in hospitals using hospitalization records from 21 district hospitals. We found variation in the gender and regional distribution of the five leading causes of hospitalization and death of children under five. Although the five causes accounted for 89% (468/523) of all neonatal hospitalizations, three causes accounted for 93% (10,851/11,632) of all-cause hospitalizations for children ages 1 to 59 months (malaria, lung disease, and acute diarrhea), malaria accounted for 69% (1086/1566) of all deaths at ages 1 to 59 months. In Burundi, human malarial infections continue to be the main cause of hospitalization and mortality among under-five children.

## 1. Introduction

The annual infant mortality rates in Burundi have decreased from 101.2 deaths per 1000 live births in 1996 to 80.8 deaths per 1000 live births in 2016 [1]. This decrease in mortality reflects specific government initiatives and policies such as performance-based financing, first implemented in 2006 as a pilot program in three provinces and scaled up in 2010 [2] to increase deliveries in health facilities and improve the quality of antenatal care [3,4,5,6]. Extensive research has shown that access to qualified staff (e.g., skilled birth attendants and nurses) during delivery can prevent maternal and early neonatal deaths [7,8,9]. In Burundi, assisted deliveries by qualified staff (nurses) increased from 76.7% in 2006 to 94.4% in 2008 in health facilities implementing performance-based financing, due to this innovative way of funding health care and services [10]. Despite the steady decrease in infant mortality over recent decades, the risk of a child dying before age five is still high in this East African country [11,12], almost 1.6 times higher than that in the World Health Organization (WHO) African region in 2016 (52.3 per 1000 live births). In some hospitals in Burundi, the neonatal mortality rate is two times higher than the national average of 4.2% [13].

When compared to the Democratic Republic of Congo (DRC), a neighboring country with similar patient settings and tormented by decades of authoritarianism, poverty and civil war, Burundi has high infant mortality rates [14]. It is also known that war influences infant mortality, [15,16,17] and Burundi is no exception. Pneumonia (19%), diarrhea (15%) and preterm birth complications (11%) [18] have been shown to be the three leading causes of deaths in Burundian children under five, while the three leading causes of morbidity have been observed to be malaria [19] followed by tuberculosis and diarrhea [20]. Studies have also shown that in conflict settings, the nature of conflict and prior conditions, weakened delivery systems, lower coverage of interventions, disempowering policies and gaps in the continuum of care continue to contribute to poor maternal, newborn, and child health outcomes [14,21,22,23,24].

Political instability and violent conflict continue to impact on maternal, newborn, and child health outcomes in contexts such as Burundi. There is a need therefore to continue documenting, analyzing and monitoring maternal, newborn, and child health outcomes to track progress towards achieving Sustainable Development Goal (SDG) 3.2. This goal aims to “end preventable deaths of newborns and children under 5 years, with all countries aiming to reduce neonatal mortality to at least as low as 12 per 1000 live births and under-five mortality to at least as low as 25 per 1000 live births” by 2030 [25]. However, although some research have been carried out on incidence and cause-specific mortality for neonatal hospitalizations [26,27,28,29,30,31,32], there is little reliable information on the specific incidence and mortality of the actual major causes of death among neonates (first month of life) and at ages 1 to 59 months (under-five years old) across sub-Saharan Africa. Thus, child health outcomes (morbidity and mortality) variations at subnational level in sub-Saharan countries remain unknown [22,33,34]. The objectives of this study were to describe the main causes of hospitalization and mortality in children during the neonatal period between 1 to 59 months, for boys and girls, in hospitals using data from 21 district hospitals.

## 2. Materials and Methods

### 2.1. Hospital Data

To assess incidence of morbidity and mortality in hospitals, hospital-based records were reviewed using the patient files, ward death and discharge registers of 21 district hospitals. A focus was placed on the major disease groups, gender, age group, area of residence, admission date, discharge date, principal and secondary diagnoses, hospital and hospital ward admitted (e.g., pediatrics) for all children aged 0 to 59 months hospitalized from 1 January 2010 through 31 December 2010. In Burundi, major disease grouping diagnosis and the probable cause of death is based on the guidelines in the Ninth Revision of the International Classification of Diseases (ICD-9-CM) [25]. As previously explained [19], malaria case definition in Burundi is based on both clinical signs (microscopy or rapid diagnostic tests (RDTs) and symptoms based on the guidelines in the ICD-9-CM, and the definition included all malaria cases (ICD-9-CM Diagnosis 084.0 to 084.9).

### 2.2. Hospitalization Rate

The age and sex-specific top five causes of the hospitalization rate were calculated as the number of hospitalizations from each of the top five causes in 2010 as reported in hospital admissions records from the 21 study hospitals, divided by the estimated national population of that age in 2008 based on the Burundi 2008 Census of Population and Housing [35]. The term “neonatal” includes infants from birth (day 0) up to and including 28 postnatal days.

The age and sex-specific all cause hospitalization rates were calculated based on the Encounter ID as described in co-authored previous studies [19,36], allowing separate admission encounters by the same patient to be linked. For instance, because a child may be treated more than once for acute diarrhea, an algorithm was developed using the Encounter ID and other identifiers (e.g., gender, address, and date of birth) so that only the first encounter in each year was used to calculate the age and sex-specific all cause hospitalization rate. Cases with incomplete records, such as dates of health facility visit, age, address, results of diagnosis, were excluded from the analysis. A complete year of data from January to December 2010 was analyzed.

### 2.3. Death Rate

As with hospitalization rates, the total number of deaths for each of the top five causes of death were obtained from the hospital admission records of the 21 study hospitals with regards to the reported deaths that required hospitalization by age group (neonates and at ages 1 to 59 months). The age- and sex-specific death rate was calculated as the number of deaths from each of the top five causes of death in 2010 divided by that age cohort’s (<1 month and at ages 1 to 59 months) estimated total hospitalizations in 2010 (denominator). Byar’s approximation of the exact Poisson distribution was then used, which is particularly precise even with small numbers [37] to calculate 95% confidence intervals for both hospitalizations and death rates.

### 2.4. Hospital Case Death Rates

To calculate the hospital death rates (HDCR), the all-cause deaths per hospital cases (including both neonates and ages 1 to 59 months) in 2010 was used, and then divided this by the number of total cases or the total number of children) who were hospitalized during the same period (2010). This number was the denominator multiplied by 100 to obtain the hospital case death rate. This denominator was used because Burundi’s census data does not provide age/sex-specific population data at the district or provincial level for political reasons [22]. For interpretation, the 21 district hospitals were partitioned into their respective provinces and the four regions of Burundi (Eastern, Northern, Southern and Western).

The hospital incidence rate was calculated based on the cases of all causes of hospitalizations for each age group (neonates and at ages 1 to 59 months) as reported in the hospital admission records. Data were collected within age groups (neonates and at ages 1 to 59 months), which were then divided by the total hospitalizations for the age group multiplied by100 children, respectively, to obtain the incidence rate per 100 child-years.

### 2.5. Ethics Statement

This study was approved by the University of Miami on 30/08/2018 (No. 20180761), with data collection collected in accordance to ethical procedures set by the Ministère de la Santé Publique et de la Lutte contre le SIDA (ethics approval number not applicable). Results were reported in an aggregated manner (at the hospital and provincial level).

## 3. Results

### 3.1. Neonatal (<1 Month) and at Age 1 to 59 Months Hospitalizations

In 2010, about 12,155 hospitalizations were reported in Burundi. Of these, 523 hospitalizations were for neonates while 11,632 hospitalizations were for children aged 1 to 59 months, and 52% of the patients were girls. All children (99%) hospitalized were from the pediatric ward. The hospitalization rate was higher for all cause hospitalizations per 1000 population (age-specific for children at age 1 to 59 months) compared to the hospitalization rate for neonates (7.6 vs. 1.8). Five causes accounted for 89% (468/523) of all neonatal hospitalizations: early neonatal infection, prematurity, fetal acute, lung disease and urinary infection, and 93% (10,851/11,632) of all hospitalizations for children ages 1 to 59 months (lung disease, malaria and acute diarrhea) (Table 1).

The proportion of under-five hospitalizations that were neonatal was higher among girls than they were among boys: early neonatal infection, prematurity, fetal acute and lung disease. For children ages 1 to 59 months, the rate of hospitalization was higher due to lung disease and malaria (includes malaria, severe malaria and pernicious malaria). Almost half (49%, 254/523) of early neonatal infection hospitalizations and nearly half (5304/11,632) of lung disease hospitalizations at ages 1 to 59 months occurred in rural hospitals.

### 3.2. Deaths during Neonatal (<1 Month) and at Age 1 to 59 Months

The all-cause mortality rate for neonates (<1 month) was 1.7 per 1000 children compared to 134.6 per 1000 children at ages 1 to 59 months (Table 2). One cause accounted for more than half (53%, 191/359) of all neonatal deaths: early neonatal infection (191 deaths, 95% confidence interval (CI) 0.014–0.019, 0.7 deaths per 1000 neonates). The all-cause mortality rate for girls at <1 month was about three times higher among girls (75%, 270/359) than boys (25%, 89/359) and most of the leading causes of death were between 28%, 19% and 7% higher in girls than boys, with the exception of urinary infection and lung disease.

Malaria accounted for 69% (1086/1566, 95% CI 0.01–0.01) of all deaths at ages 1 to 59 months. In this age group, the all-cause mortality rate was about 19% higher among girls (59%, 928/1566) than boys (41%, 628/1566) and most of the leading causes of death were between 13% and 30% higher in girls than they were among boys, with the exception of malaria (severe pernicious malaria) and acute diarrhea.

Rural hospitals accounted for a greater proportion of all-cause deaths (32% (148/359) for neonatal deaths and 41% (508/1566) for children ages 1 to 59 months (Table 1 and Table 2).

### 3.3. Hospital Variation in Incidence and Mortality Rates

The hospital and regional distribution of hospitalizations and deaths from all causes varied among neonates and at ages 1 to 59 months (Table 3). In the hospitals located in northern Burundi, the all-cause hospitalizations represented 62% (range 1–20%) of the total cases, compared to neonates, where cases of hospitalization represented 4% (range 1–24%) of total cases. For neonates, the range of the hospital case death rate (percent) was from a low of 0.19 in Matana hospital located in southern Burundi to a high of 17.02% in Kirundo hospital located in the northern part of the country.

## 4. Discussion

An initial objective of this study was to describe the main causes of hospitalization and mortality in children during the neonatal period and at ages 1 to 59 months, for boys and girls, and the total annual burden of neonatal and child under-five morbidity and mortality in each district using data from 21 district hospitals in Burundi. The study found that malarial infections are the leading cause of mortality in Burundi, causing three-quarters of all 1,566 child deaths in 2010 (1086 of 1566 deaths). The main factors contributing to malaria morbidity and mortality in Burundi include “climate change, population density, shifting agricultural practices, food insecurity and a lack of prevention awareness” [38]. This finding accords with the author’s previous co-authored study, which showed that population density increased the risk of malaria exposure, with most cases of malaria in children found at the higher elevations of the northern parts of Burundi [19]. This suggests that malaria-transmitting mosquitoes are finding higher elevations more livable today because these areas are becoming warmer and wetter. This finding also corroborates earlier findings in other settings [39,40,41,42]. At the same time, prolonged conflict in the country has led to the significant annihilation and disruption of the healthcare system, services and the quality of health including insufficient human, financial and logistical resources, leading to increased inaccessibility of healthcare for many Burundians [22,43,44]. Improvements in healthcare infrastructure and delivery, and a better understanding of the multiple environmental and human population influences on malaria transmission can improve prevention efforts, the malaria control response and would reduce child deaths and narrow the gender gap in child mortality in Burundi [45].

The marked provincial/regional differences in all-cause mortality among neonates must reflect the quality of midwifery, obstetric, and pediatric care available [22,46]. However, girls in Burundi die more commonly from the top five causes than their male counterparts and disparities in access to care, not genetic or biologic determinants, are a more possible explanation of these observed gender differences. These results match those observed in earlier studies [34]. In addition, and consistently with the literature [20], this study found no gender differences regarding the incidence of diarrhea. These results are likely to be related to the supportive environment deliberately put in place by the Burundian Government in collaboration with partners to aggressively reduce infant and child mortality rates. For example, in 2004, the government of Burundi institutionalized output-based financial support or performance-based financing (PBF) of the facilities [22]. Performance-based financing has shown to be a promising reform approach to improving the utilization, the quantity and quality of maternal and child health services in Burundi and in neighboring countries such as Rwanda [3,10,47,48]. Other effective intervention programs for the management of childhood diarrhea have been those to do with the “social marketing intervention that promoted the Population Services International (PSI) ORASEL Kit, a low osmolarity oral rehydration salt (ORS), a product developed by FDC limited (http://www.fdcindia.com/index.php). This ORS is branded and distributed by PSI” [49] as well as other hygiene education and point-of use household water treatment interventions implemented in the country [20].

This study found that four of the five district hospitals in the current study (Bururi, Gitega, Kirundo, Makamba and Ruyigi) were part of the initial rollout of PBF schemes [10], and have a hospital case death rate (percent) that ranges from 4.59 (Gitega hospital) to 17.07 (Kirundo district hospital). Further research is needed to understand the progress made and persisting challenges, specifically in regards to the impact of PBF on infant and child mortality and inequities. This study further suggests that specific interventions might be a priority for different age groups in different regions in the primary and tertiary prevention phases (for example, preventive strategies to reduce the burden of lung disease at ages 1 to 59 months or early neonatal infection would be particularly needed in Eastern, Northern and Southern Burundi). The government of Burundi should also continue to monitor the top causes of morbidity and mortality to reduce child mortality in the country.

A limitation of this study is its cross-sectional design and that the data was collected only from major district hospitals. However, given the specific study objective, the study design is not a problem. The use of the incidence rate, a more precise measure, adds a better estimate of the true at-risk population in this etiological research. In addition, given that the census data for Burundi do not provide age-specific population data for political reasons, it is not possible to discern the true at-risk population of children at the provincial or district level. This fact limits the interpretation of the data, particularly to the actual denominator of the general population at risk. This limitation was addressed by calculating the incidence rate based on the total hospitalization cases at each district hospital divided by the total for all causes of hospitalizations for each age group (>1 month and at age 1 to 59 months) as reported in hospital admissions records. Finally, further research into the cause and etiology of child diseases across Burundi would significantly benefit our understanding of the nature of the causes of morbidity and mortality in children in Burundi.

## 5. Conclusions

In summary, there was variation in the gender and regional distribution of the five leading causes of child hospitalizations and deaths among neonates and at ages 1 to 59 months in Burundi. Malarial infections caused three-quarters of all 1566-child deaths in 2010. This study’s findings suggest that improved maternal and perinatal outcomes may be achieved by improving prevention efforts, malaria control programs and that the identification and understanding of the influence of multiple environmental and human population influences on malaria transmission would reduce child deaths and narrow the gender gap in child mortality Burundi. There is, therefore, a definite need to develop or enhance specific regional and age group interventions/policies at the primary and tertiary prevention phases.

## Figures and Tables

**Table 1:** Causes of hospitalization in neonates (<1 month) and at age 1 to 59 months in Burundi, 2010.

Causes	Girls	Boys	Total (95% CI)	Rural Area	* Rate per 1000
**Neonate hospitalizations**					
Early neonatal infection	177	77	254 (0.427–0.548)	95	0.9
Premature	83	10	93 (0.144–0.217)	52	0.3
Suffering fetal acute	38	15	53 (0.077–0.131)	1	0.2
Lung disease	25	17	42 (0.0587–0.107)	11	0.1
Urinary infection	10	16	26 (0.033–0.072)	9	0.1
All causes (total)	350	173	523	192	1.8
**1 to 59 months hospitalizations**					
Lung disease	2812	2492	5304 (0.444–0.468)	2042	3.4
Malaria	2576	2617	5193 (0.03–0.04)	1310	3.4
Acute diarrhea	154	200	354 (0.027–0.037)	107	0.2
All causes (total)	5965	5667	11,632	3696	7.6

A total of 47 of the neonate cases did not have a diagnostic code and thus were not included when calculating the hospitalization rate. * The age-specific hospitalization rate was calculated as the number of hospitalizations from each of the top five causes in 2010 divided by that age cohort’s (<1 month and at ages 1 to 59 months) estimated national population in 2008 per 1000 population. CI: confidence interval.

**Table 2:** Causes of death in neonates (<1 month) and at age 1 to 59 months in Burundi, 2010.

Causes	Girls	Boys	Total (95% CI)	Rural Area	* Rate per 1000 Children
**Neonatal deaths**					
Early neonatal infection	145	46	191 (0.014–0.019)	79	0.7
Premature	76	7	83 (0.005–0.009)	51	0.3
Suffering fetal acute	33	9	42 (0.003–0.005)	1	0.1
Urinary infection	9	13	22 (0.001–0.003)	8	0.1
Lung disease	2	2	4 (0.000–0.001)	0	0.0
All causes of deaths (total)	270	89	359	148	1.2
**1 to 59 months deaths**					
Malaria	641	445	1086 (0.01–0.01)	354	0.6
Lung disease	97	55	152 (0.008–0.011)	52	0.4
Acute diarrhea	50	84	134 (0.003–0.006)	48	0.2
All causes of deaths (total)	928	638	1566	508	134.6

* The age-specific death rate was calculated as the number of deaths from each of the top five causes of deaths in 2010 divided by that age cohort’s (<1 month and at ages 1 to 59 months) estimated total hospitalizations in 2010 per 1000 population.

**Table 3:** All causes of hospitalization and death rates by age, region and province, 2010.

District Hospital	Neonates	At Ages 1 to 5 Months	Province/Region
Gisuru			Ruyigi/Eastern
Cases	1	1	
Incidence ^a^	0.19	0.01	
HCDR (%) ^b^	~	~	
Kinyinya			Ruyigi/Eastern
Cases	1	1	
Incidence ^a^	0.00	0.01	
HCDR (%) ^b^	~	~	
Ruyigi Rural			Ruyigi/Eastern
Cases	1	1	
Incidence ^a^	0.00	0.01	
HCDR (%) ^b^	~	~	
Gitega			Gitega/Eastern
Cases	28	1261	
Incidence ^a^	5.35	10.84	
HCDR (%) ^b^	4.59	1.07	
Mutaho			Gitega/Eastern
Cases	11	323	
Incidence ^a^	2.10	2.78	
HCDR (%) ^b^	1.91	0.46	
Kanyaza			Kayanza/Northern
Cases	66	1399	
Incidence ^a^	12.62	12.03	
HCDR (%) ^b^	0.57	0.07	
Kirundo			Kirundo/Northern
Cases	127	1692	
Incidence ^a^	24.283	14.55	
HCDR (%) ^b^	17.02	3.19	
Mukenke			Kirundo/Northern
Cases	40	1348	
Incidence ^a^	7.65	11.59	
HCDR (%) ^b^	4.78	1.82	
Musema			Kayanza/Northern
Cases	9	169	
Incidence ^a^	1.72	1.45	
HCDR (%) ^b^	1.15	0.52	
Muyinga			Muyinga/Northern
Cases	5	2377	
Incidence ^a^	0.96	24.44	
HCDR (%) ^b^	0.57	1.34	
Nyanza Lac			Muyinga/Northern
Cases	36	548	
Incidence ^a^	6.88	4.71	
HCDR (%) ^b^	4.21	0.83	
Bururi			Bururi/Southern
Cases	58	582	
Incidence ^a^	11.09	5.00	
HCDR (%) ^b^	8.41	0.36	
Matana			Bururi/Southern
Cases	6	196	
Incidence ^a^	1.15	1.69	
HCDR (%) ^b^	0.19	0.09	
Makamba			Makamba/Southern
Cases	12	485	
Incidence ^a^	2.29	4.17	
HCDR (%) ^b^	16.06	0.07	
Cibitoke			Cibitoke/Western
Cases	20	169	
Incidence ^a^	3.06	1.45	
HCDR (%) ^b^	3.82	0.64	
Mabayi			Cibitoke/Western
Cases	36	83	
Incidence ^a^	6.88	0.71	
HCDR (%) ^b^	6.69	0.52	
Ijenda			Bujumbura Rural/Western
Cases	10	240	
Incidence ^a^	1.91	2.06	
HCDR (%) ^b^	1.91	0.12	
Rushubi			Bujumbura Rural/Western
Cases	7	203	
Incidence ^a^	0.013	0.02	
HCDR (%) ^b^	1.15	0.12	
Rwibaga			Bujumbura Rural/Western
Cases	3	338	
Incidence ^a^	~	0.03	
HCDR (%) ^b^	~	~	
Kiganda			Muramvya/Western
Cases	12	211	
Incidence ^a^	2.29	1.81	
HCDR (%) ^b^	1.53	0.28	
Muramvya	2	2	Muramvya/Western
Cases	~	~	
Incidence ^a^	0	0.00	
HCDR (%) ^b^	~	~	

^a^ Incidence per 1000 children years. ^b^ Hospital case death rate (percent). ~ no case was reported. HCDR: hospital case death rate.
